# Gastrointestinal Endoscopic Outcome in Late Adolescent Women With Iron-Deficiency Anemia in Basrah-Iraq: A Multicenter Study

**DOI:** 10.7759/cureus.14630

**Published:** 2021-04-22

**Authors:** Samih A Odhaib, Miaad J Mohammed, Saad S Hammadi

**Affiliations:** 1 Adult Endocrinology, Faiha Specialized Diabetes, Endocrine and Metabolism Center, College of Medicine, University of Basrah, Basrah, IRQ; 2 Diagnostic Radiology, Al-Refaee General Hospital, Thi-Qar Health Directorate, Thi-Qar, IRQ; 3 Internal Medicine, College of Medicine, University of Basrah, Basrah, IRQ

**Keywords:** adolescents, basrah, endoscopy, iraq, iron deficiency anemia, gastrointestinal, symptoms

## Abstract

Background

Iron deficiency anemia (IDA) in late adolescent women has multiple pathophysiologies. Silent blood loss, celiac disease (CD), malignancies, and other gastrointestinal (GI) lesions receive much attention during IDA management. There is no consensus about endoscopic screening. Our study evaluates factors affecting GI endoscopic diagnosis for the etiology of IDA in late adolescent women.

Materials and Methods

We conducted an observational, multicenter retrospective analysis of 192 adolescent women with IDA admitted for GI endoscopic diagnosis from 2006 to 2016. Baseline measurements included hemoglobin, serum ferritin, mean corpuscular volume, serum iron, total iron-binding capacity, and transferrin saturation. We collected demographic characteristics, duration of hospital stay, the degree of severity of anemia, and endoscopic findings.

Results

The mean age was 19±1 years (range 17 to 21 years), with mildly to moderately severe IDA. Patients received esophagogastroduodenoscopy (EGD, n=178) or colonoscopy (n=14). The mean hospital stay was 2.0±1.0 days. We found negative endoscopies (n=74), CD (n=85), gastric ulcer (n=19), malignancy (n=2), inflammatory bowel disease (n=1), and other nonsignificant endoscopic findings (n=11). We found no correlation between the duration of the hospital stay with the severity of IDA, no significant association between GI symptoms of the patients with endoscopic findings, and a significant but weak association between GI symptoms and serum ferritin.

Conclusions

In late adolescent women with IDA who have significant GI endoscopic lesions, the GI symptoms are of limited value in guiding the endoscopic diagnostic approach for evaluation of IDA.

## Introduction

According to the World Health Organization (WHO), adolescence is a developmental phase of change from the pediatric stage to young puberty, then to a sexually mature one with a different mental and adult identity. The WHO divides adolescence into early (10-13 to 14-15 years), middle (14-15 to 17 years), and late (17-21 years) adolescence, with a clear overlap between the three categories [1].

Adolescents (especially women) have a high risk of developing iron-deficiency anemia (IDA) due to physiological and pathological factors [2]. Iron requirements peak during adolescence when compared to younger age groups due to rapid pubertal growth, including a sharp increase in body mass, blood volume, and red cell mass, which increases iron demand two to three-fold from a pre-adolescent level. Iron requirements will increase further after sexual maturation and pregnancy, exacerbating iron loss for which a dietary source is not enough for replenishment [2-5].

Recent changes in dietary habits may contribute to the development of IDA in adolescents; because of this, they will not meet their iron requirements due to growth spurts, especially in adolescent females, given their regular menstrual blood loss [3]. Other contributory factors include erratic eating habits, food aversion for iron-rich items like green leafy vegetables, and encouraging iron absorption inhibitors in tea and coffee (phytates/tannins) [6].

The onset of pregnancy during adolescence further increases demands for iron and aggravates iron deficiency (ID) [2,5] and thereby contributes to poor maternal and fetal outcomes later on [2].

The frequent occurrence of infectious diseases and parasitic infestation of this age group further increases requirements for iron and increases the chances for a negative iron status and IDA [2,3]. Recent studies proposed a link of questionable significance between *Helicobacter pylori* infection with IDA in different age groups [7,8].

Iron deficiency anemia in late adolescent women has multiple pathophysiologies. Silent blood loss, celiac disease (CD), malignancies, and other gastrointestinal (GI) lesions receive the most attention during management; there is no consensus about endoscopic screening [8-10].

The current guidelines do not recommend GI endoscopic screening for young adolescent females, except for case-by-case considerations in a symptom-directed approach [11,12]. However, the GI symptoms of IDA in adolescent females may be atypical, unlike those in adults and the pediatric age group [8,13].

In this study, we attempted to assess the factors affecting GI endoscopic outcomes in late adolescent women with IDA.

## Materials and methods

This observational study involved the retrospective analysis of the medical records of 192 adolescent women who presented to Al-Sadr Teaching Hospital, Faiha Teaching Hospitals, and Basrah Oncology and Hematology Center for diagnosing the etiologies of IDA from January 2006 to January 2016. Medical record data collected were not electronic as the collection period occurred before full automation of the records. We evaluated data from endoscopy, laboratory, histopathology, and hospital admission units.

**Figure 1:**
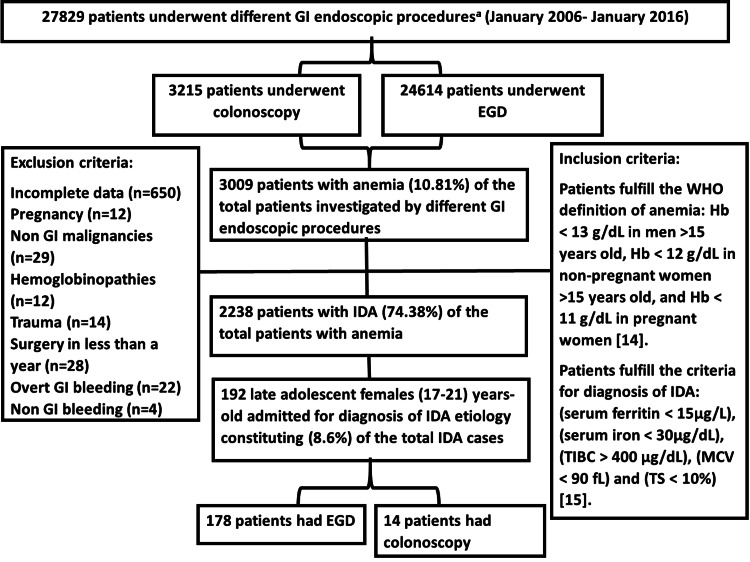
Flow chart of data collection during the study of 192 late adolescent women admitted for investigation of the cause of iron deficiency anemia. BDE, bidirectional endoscopy; EGD, esophagogastroduodenoscopy; GI, gastrointestinal; Hb, hemoglobin; IDA, iron deficiency anemia; MCV, mean corpuscular volume; TIBC, total iron-binding capacity; TS, transferrin saturation; WHO, World Health Organization. ^a^ All GI endoscopic procedures utilized the Pentax Medical (HOYA Group, Tokyo, Japan) or Olympus (Olympus Surgical Technologies America, Southborough, MA, USA) endoscopic system.

Figure 1 illustrates the process of data collection, the inclusion and exclusion criteria, and the selection of the patients for final analyses, which followed strict inclusion criteria based on Odhaib et al. and Kepczyk et al. [14,15].

All patients enrolled had measurements of their hemoglobin (Hb), serum ferritin, mean corpuscular volume (MCV), serum iron, total iron-binding capacity (TIBC), and transferrin saturation (TS). We tabulated the data according to the following

A) Demographic characteristics.

B) Duration of hospital stay.

C) Analysis of the GI symptoms:

● Upper GI symptoms: dysphagia, heartburn, nausea, vomiting, upper abdominal pain (and its relief by food or antacids).

● Lower GI symptoms: changed bowel habit, diarrhea, constipation, and colicky lower abdominal pain.

● Asymptomatic was the term used to describe IDA patients without GI symptoms.

● Mixed symptoms: the patient had upper and lower GI symptoms together.

D) Different GI endoscopic findings.

E) Celiac serological tests.

F) The severity of IDA [15]:

● Severe IDA when Hb ˂ 9 g/L, serum ferritin ˂ 9 µg/L, MCV ˂ 70 fL, serum iron ˂ 15 µg/dL, TIBC ˃ 400 µg/dL, or TS ˂ 3.5%.

● Mild to moderate IDA when Hb 9 to less than 13 g/L, serum ferritin 9-15 µg/L, MCV 70-90 fL, serum iron 15-30 µg/dL, TIBC 360- 400 µg/dL, or TS 3.5-10%.

Patients were diagnosed with biopsy-proven celiac disease (CD) if an endoscopic biopsy revealed Marsh type 3 (A, B, and C), according to the American College of Gastroenterology clinical guidelines [11]. The main concern focused on type 3, in which there are crypt hyperplasia, increased intraepithelial lymphocytes, along with partial villous atrophy in type 3A, subtotal villous atrophy in type 3B, and total villous atrophy in type 3C. The estimation of the anti-tissue transglutaminase IgA (ATTGA) and IgG (ATTGG) was done using enzyme-linked immunosorbent assay (ELISA) (BioTek ELx800, USA, and Alegria® by ORGENTEC Diagnostika, Germany). Values > 10 U/mL for ATTGA and > 9 U/mL for ATTGG were considered positive. Values < 4 U/mL for ATTGA and < 6 U/mL for ATTGG was considered negative. The values between the positive and negative results were considered weak positive.

Data were entered and matched via Microsoft Access and Excel (Microsoft Corporation, Redmond, WA, USA) and then analyzed on IBM SPSS Statistics for Windows, Version 26.0 (IBM Corp., Armonk, NY, USA). The study used the mean ± standard deviation or frequency (%) for data expression. To compare and correlate the means, we used bivariate correlation analysis. The study considered a two-tailed P-value ≤ 0.05 to be statistically significant.

## Results

There were 192 late adolescent female patients with IDA of mild to moderate severity. These patients represented 8.6% of the total inpatients with IDA during the 10 years of study. The mean age was 18.85±1.22 years (Figure 1 and Table 1).

**Table 1:** The demographic characteristics of 192 late adolescent women admitted for investigation of the cause of iron deficiency anemia. GI, gastrointestinal, IDA: iron deficiency anemia, Ig: immunoglobulin. ^a^ All positive cases were Marsh type 3, and positive for ATTGA, and 88% (n=75) were ATTGG positive.

Parameters		Value
Age in years (mean ± standard deviation)		18.85±1.22
Celiac disease diagnosis	Referred for celiac disease (%)	154 (80.2)
	Anti-tissue transglutaminase antibody IgA positive	91 (47.4)
	Anti-tissue transglutaminase antibody IgG positive	90 (46.9)
	Biopsy-proven celiac disease (%)^a^	85 (44.3)
	Age in years (mean ± standard deviation)	18.84±1.28
IDA parameters (mean ± standard deviation)	Mean hemoglobin (g/L)	10.54±0.66
	Mean ferritin (µg/L)	12.67±1.65
	Mean corpuscular volume (fL)	76.41±8.45
	Mean serum iron (µg/dL)	25.11±3.94
	Mean total iron-binding capacity (µg/dL)	375.21±15.28
	Mean transferrin saturation%	6.70±1.06

There were 154 adolescent women who were referred for possible CD diagnosis, out of whom 85 women had a definite diagnosis of CD. The mean age of adolescents with CD and the mean age of the overall cohort were similar.

**Figure 2:**
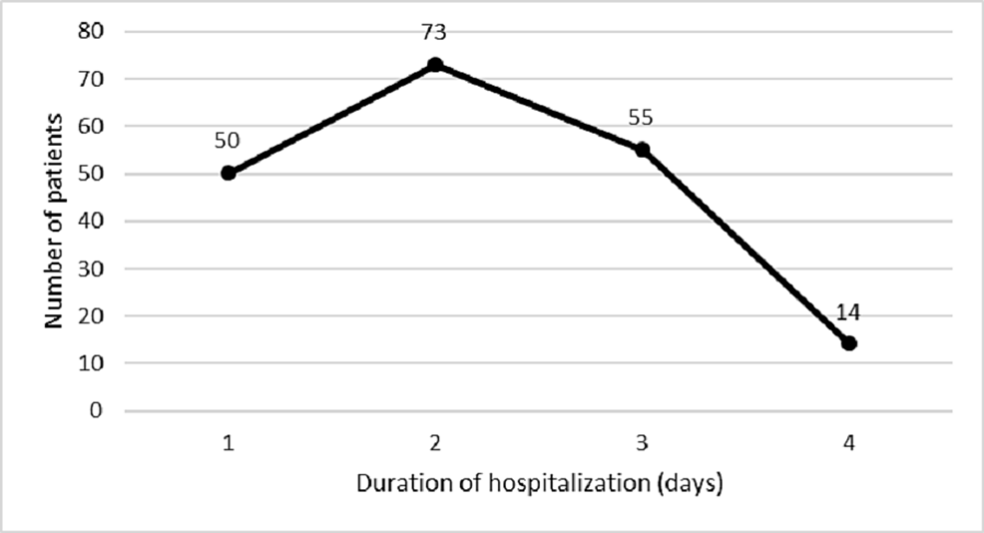
The duration of hospitalization of 192 late adolescent female patients who underwent different gastrointestinal endoscopic procedures for diagnosis of iron-deficiency anemia etiology. The mean duration of hospitalization 2.0±1.0 days. There was no correlation to any parameter of iron deficiency anemia with the length of hospital stay (P > 0.05) by bivariate correlation analysis.

The maximum duration of hospital stay was four days, with a mean duration of (2.0±1.0) days (Figure 2). There was no significant relationship to any parameter of IDA with the length of hospital stay (P > 0.05) by bivariate correlation analysis.

**Figure 3:**
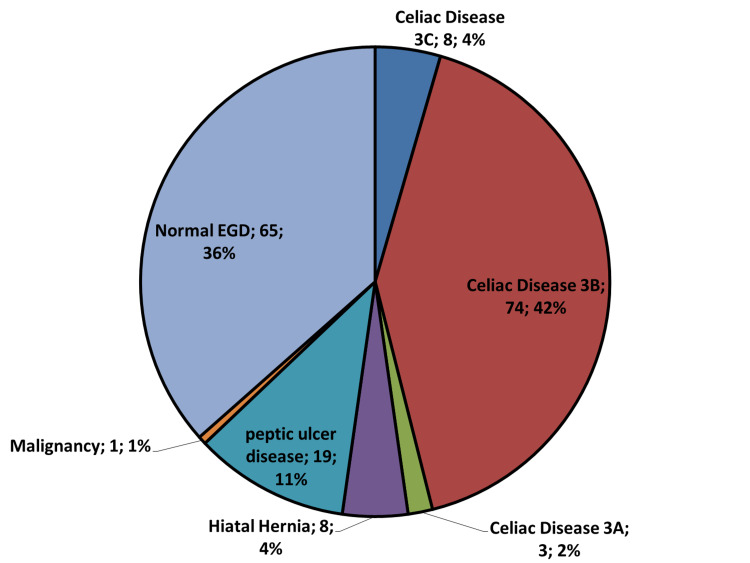
Pie chart of different upper gastrointestinal endoscopic findings of 178 late-adolescent women with iron deficiency anemia who underwent esophagogastroduodenoscopy for diagnosis of the iron-deficiency anemia etiology. EGD, esophagogastroduodenoscopy; GI, gastrointestinal; IDA, iron deficiency anemia.

**Figure 4:**
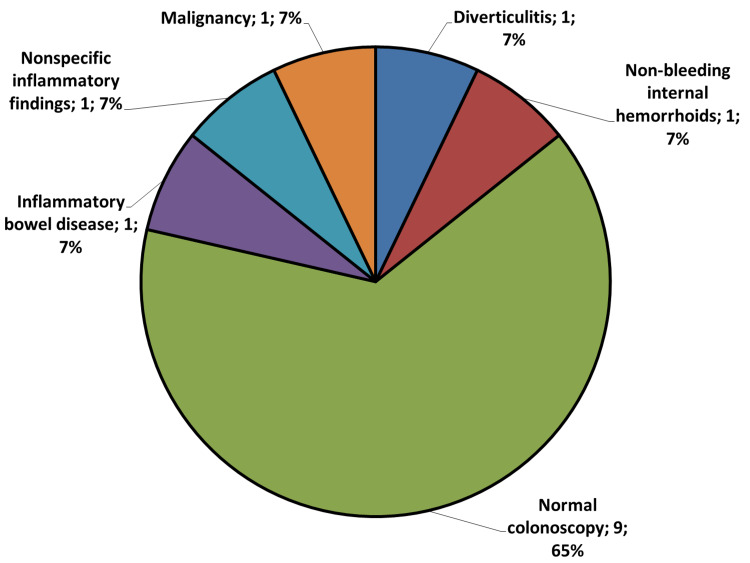
Pie chart of different upper gastrointestinal endoscopic findings of 14 late-adolescent female patients who underwent colonoscopy for diagnosis of the iron-deficiency anemia etiology. Nine patients had a negative colonoscopy, with the other lesions in one patient each. IBD, inflammatory bowel disease; GI, gastrointestinal; IDA, iron deficiency anemia.

Figures 3 and 4 demonstrate the different GI endoscopic findings for the 192 late adolescents in our study. All the endoscopic findings were considered significant except for negative endoscopic studies, hiatal hernia, nonbleeding internal hemorrhoids, and nonspecific colonic inflammatory changes.

As shown in Table 2, we found no relevant association between GI symptoms of the patients with pathological endoscopic findings and a significant but weak association between GI symptoms and serum ferritin.

**Table 2:** The association between the symptomatology with possible pathological endoscopic lesions in 108 adolescent women with iron deficiency anemia. GI, gastrointestinal; N/A, not applicable. ^a^ Excludes hiatal hernia and normal or negative esophagogastroduodenoscopy. ^b^ Excludes nonspecific inflammatory changes, nonbleeding hemorrhoids, and negative colonoscopy.

Parameters	Upper GI symptoms n=56 (%)	Lower GI symptoms n=36 (%)	Asymptomatic n=12 (%)	Mixed symptoms n=4 (%)	P
Significant upper GI findings (n=105)^a^	56 (53.33)	33 (31.43)	12 (11.43)	4 (3.81)	0.905
Significant^2^ colonoscopic findings (n=3)^b^	0	3 (100)	0	0	N/A
Malignancy diagnosis (n=2)	1 (50)	1 (50)	0	0	N/A
Biopsy-proven celiac disease (n=85)	45 (52.94)	27 (31.76)	9 (10.59)	4 (4.71)	0.667
Serum ferritin ≥ 9 µg/L (n=107)	56 (52.34)	36 (33.64)	11 (10.28)	4 (3.74)	0.044
Hemoglobin 9 to < 13 g/L (n=108)	56 (51.85)	36 (33.34)	12 (11.11)	4 (3.70)	N/A
Mean corpuscular volume 70-90 fL (n=99)	53 (53.54)	32 (32.32)	11 (11.11)	3 (3.03)	0.481
Serum iron 15-30 µg/dL (n=108)	56 (51.85)	36 (33.34)	12 (11.11)	4 (3.70)	N/A
Total iron binding capacity 360-400 µg/dL (n=106)	56 (52.83)	35 (33.02)	11 (10.38)	4 (3.77)	0.253
Transferrin saturation 3.5-10% (n=108)	56 (51.85)	36 (33.34)	12 (11.11)	4 (3.70)	N/A

## Discussion

Iron deficiency anemia in women of reproductive age represents a diagnostic challenge given their possible menstrual and pregnancy-associated iron loss [10]. Some reports consider reduced iron absorption and insidious blood loss of variable etiology from the GI tract as the most frequent causes of IDA in adolescents [10,16]. Although anemia in adolescent women is a common finding, there is insufficient evidence to recommend universal laboratory screening for ID in nonpregnant adolescent women. There is no national study that evaluates GI endoscopy in IDA patients in general, and adolescents specifically.

The most importantly encountered GI lesion was biopsy-proven CD found in 85 adolescents out of 154 inpatients referred for possible diagnosis of CD. This constituted 3.4% of the total 2238 patients with IDA who underwent endoscopic diagnosis during that period. Our finding is more than that of Nahon S study [17], and the figure in the British consensus guidelines, 2-3% [11], likely due to the variable local prevalence of CD and different inclusion criteria. All the positive cases were ATTGA positive, while 88% (n=75) were ATTGG positive.

Despite the high prevalence of CD, it is often unrecognized and under-investigated in patients presenting with IDA [10,17]. Highly sensitive and specific laboratory tests aid distal duodenal biopsy in achieving a definite diagnosis for CD in a large proportion of the population with dyspeptic symptoms [9,17,18]. Many studies gave controversial opinions about the usefulness of duodenal biopsies during endoscopy if no apparent cause of IDA could be found [11] or negative serology [9] or lack of any GI symptoms [19,20].

Serological testing before endoscopy can be cost-effective in selecting at-risk women or patients with silent CD for EGD. However, the sensitivity of these antibodies does not approach 100%, and the diagnostic accuracy varies dramatically between laboratories; therefore, reliance solely on serology may potentially miss the occasional patient with CD [9,17]. It is crucial to confirm the diagnosis of CD in every young premenopausal woman with IDA because it is a potentially treatable condition, and such diagnosis will avert other unnecessary invasive investigations for IDA [10,17,21].

The duration of hospitalization for adolescent women in the study ranges from one to four days with an approximate mean of 2±1 days; this did not correlate with any parameter for IDA. Unfortunately, there are no studies at present that deal with the effect of this factor.

Following previous studies [22,23], we considered negative endoscopic results, hiatal hernia, non-bleeding hemorrhoids, and nonspecific colonic inflammatory lesions as unlikely endoscopic causes for the development of IDA.

A comparison of positive findings in 56% of the adolescents in our cohort with those of other published papers [10,24-26] revealed that the percentage of pathologic lesions in EGD varies considerably, from 13% to 55%, due to the difference in inclusion criteria and study design set by other authors.

Fireman et al. considered GI endoscopic findings in premenopausal young women with IDA to be less serious and reported no significant difference when compared to older patients [18], observing a similar pattern of anemia in young and old age groups.

According to the European consensus on the diagnosis and management of IDA in inflammatory bowel disease (IBD), physicians should seek IDA in patients with IBD. The anemia in IBD is either IDA, anemia of chronic disease, or anemia of mixed origin [23]. Chronic IBD is considered a positive finding and a possible cause for IDA. Anemia is the most common extraintestinal complication of chronic IBD, due to defective iron absorption, decreased iron release from iron stores, and quiescent GI blood loss [22]. For patients with a negative endoscopy who remain undiagnosed, the lack of a widely disseminated coherent picture about the diagnostic sequence for IDA may be one of the chief barriers to prevent its proper management.

There was no association between the different GI symptoms with the possible GI findings or with the degree of anemia. The only significant association was with ferritin level (9-15 µg/L). The lack of association was evident in many studies [10,17,20,24,27]. Older studies suggested that the classic presentation tends to be rare, with a shift towards milder symptoms in adults and children. Sometimes, isolated IDA is the only manifestation of hidden, related GI pathology. Despite visible mucosal lesions, the disease can be even symptom-free and clinically silent, especially in diseases like CD [28] where GI symptoms alone cannot accurately differentiate CD from other common GI disorders [9].

There are conflicting reports about the importance of the presence of GI symptoms as a predictor for possible pathological endoscopic lesions [5,19,20]. Additional predictors reported by Vannella et al. were Hb, ferritin, and MCV [19], though these were not proven in our study.

This study had limitations, such as the retrospective design that harbors possible inherent referral bias since all patients studied were selected only after being referred for endoscopy. Additionally, as with other observational studies, we cannot differentiate the association from causality. From the diagnostic point of view, we do not have any patients with Marsh 1 or 2. Also, we could not find data about the utility of endomysial antibodies or *H. pylori *evaluation before 2016.

## Conclusions

Anemic adolescent women constituted 8.6% of the total IDA cases encountered during our 10-year observation, with CD as the most commonly encountered endoscopic pathology. The endoscopic diagnosis did not rely on a symptoms-led approach. The presence of GI symptoms did not have any significant association with the endoscopic pathologies or the severity of IDA, and it is of little value in directing the GI endoscopic studies in late adolescent women with IDA. Serum ferritin > 9 µg/L had a significant but weak association with the GI symptoms in patients with pathological GI lesions.

Finally, late adolescent women can benefit from a more comprehensive diagnostic protocol, a cost-effective clinical strategy for the management of IDA. Reevaluation of the clinical guidelines is needed to draw a roadmap for a universal diagnostic approach for IDA in non-pregnant women.
